# Comparison of hock- and footpad-injection as a prostate adenocarcinoma model in rats

**DOI:** 10.1186/s12917-018-1659-x

**Published:** 2018-11-06

**Authors:** Henning Richter, Agnieszka Karol, Katja Nuss, Aymone Lenisa, Erika Bruellmann, Stella-Saphira Maudens, Heinrich Hoffmann, Brigitte von Rechenberg, Patrick R. Kircher

**Affiliations:** 1 Clinic of Diagnostic Imaging, Vetsuisse Faculty, University of Zurich, Winterthurerstrasse 260, 8057 Zurich, Switzerland; 2 Musculoskeletal Research Unit, Zurich, Switzerland; 3 Center for Applied Biotechnology and Molecular Medicine, Vetsuisse Faculty, University of Zurich, Winterthurerstrasse 260, 8057 Zurich, Switzerland; 4 Philips AG, Allmendstrasse 140, 8027 Zürich, Switzerland; 5 Powder Technology Lab, IMX_LTP, Station 12, MXD 340, EPFL, 1015 Lausanne, Switzerland

**Keywords:** Footpad injection, Hock injection, 3R, Tumor model, MatLyLu, Copenhagen rat

## Abstract

**Background:**

Objective of this study is a feasibility-test comparing hock- and footpad-injection in rats with inoculated MatLyLu - adenocarcinoma tumor model. This study compares the development of an adenocarcinoma model (MatLyLu) in 12 Copenhagen rats. Two groups (*n* = 6) of animals were inoculated with 1 × 10^6^ MatLyLu tumor cells solved in 0.1 ml NaCl either by footpad or hock injection. All animals were examined before tumor inoculation and before euthanasia using a 3.0 Tesla MRI. Histological evaluation of all organs was performed post mortem.

**Results:**

Both types of injection were able to induce the adenocarcinoma model using MatLyLu tumor cells. The primary tumor could be visualized in MRI and confirmed histologically. Comparing the risk of reflux and the maximum injection volume during injection, the hock injection was superior to the footpad injection (less reflux, less anatomical restrictions for larger volumes). The hock injection induces a faster tumor growth compared to the footpad injection. As consequence the maximum level of long term discomfort after hock injection was reached earlier, even if it grew on a not weight bearing structure. Early lymph node tumor metastasis could not be observed macroscopically nor detected histologically. Therefore the reproducibility of the MatLyLu tumor model is questionable.

**Conclusion:**

Hock injection is a feasible alternative technique compared with footpad-injection in rats. It provides a save and easy injection method for various early-terminated applications with the potential to increase animal welfare during tumor models in rats.

**Electronic supplementary material:**

The online version of this article (10.1186/s12917-018-1659-x) contains supplementary material, which is available to authorized users.

## Background

Prostate cancer in human varies in differentiation and pathology, as well as in responsiveness to treatment. Prostate cancer is a common cancer in men in Europe with the incidence of clinically diagnosed patients in northern and western Europe > 200 per 100,000 men/year. [1] Beside some rare types of neuroendocrine prostate cancers (small or large cell prostate cancer), the most common type is the adenocarcinoma, with its origin at the glandular epithelial. In the following, the term prostate cancer is used for the adenocarcinoma type. Diagnostic methods are based on PSA (Prostate Specific Antigen) screening and digital rectal examination (DRE) with subsequent transrectal ultrasound guided (TRUS)-guided biopsy. Prostate cancer is suspected on the basis of these examinations, but the definitive diagnosis depends on histopathological verification. [1]

Localized or locally advanced prostate cancer has 3 major treatment options: observation, surgery and radiation. There are other nonstandard treatment options, which include cryotherapy, high-intensity focused ultrasound, and primary hormone therapy. Choosing the best treatment is generally based on the patient’s age, the stage and grade of the cancer, the general health, and evaluation of the risks and benefits of each therapy option.

A strong need exists for a reliable animal model of prostate cancer that reflects different tumor stages and can be translated into human patients to study several therapeutic approaches. An animal model needs to be based on proper experimental design and should increase the understanding of the biology of this disease. From all established rodent models of prostate cancer the Dunning model is well described and characterized by rapid growth of primary tumor and production of metastases. [2] The Dunning model has been obtained from Dunning prostate tumor-bearing Copenhagen rats on the strongly metastatic MAT-LyLu cell line. The latter can be transplanted and transfected. Histologically the tumor is characterized as an undifferentiated anaplastic form, proves invasive and fast growing as well as spreading into surrounding tissue. [3] There are different methods described for developing a locally advanced orthotopic primary tumor based on percutaneous injection of the cells in the area of interest. [4] Footpad injection is a commonly used immunization method in mice, alternatively the hock injection has been utilized as less painful considering the weight-bearing structures. [5] To the best of the authors knowledge hock injection has never been tested in rats for induction of tumors before. Considering the animal size, hock injection in rats could be beneficial for the general macroscopical and radiological screening. It would also facilitate the amount of tissue obtained for processing. Metastatic involvement of the injection-site associated tumor can be estimated through examination of lymphatic components. Undertaken the pattern of lymphatic drainage in adult laboratory rats [6] the metastatic activity is expected to be primarily in popliteal and inguinal lymph nodes in case of proximal metastases and secondarily in pulmonary lymph nodes and other organs. Histological and radiological nodal staging with detecting possible metastases is a routine method in medical diagnostics.

The aim of this study was to evaluate the feasibility of two inoculation methods (footpad versus hock injection) of an adenocarcinoma cells (MatLyLu) in Copenhagen rats and to evaluate both using a specially developed score sheet for animal health (Additional file 1). The examination included tumor behavior, tumor biology and metastatic potential using an appropriate image guidance (MRI) and histological characterization. Furthermore we aimed to compare these two methods considering the general animal welfare. The hypothesis was, that the hock injection is an alternative method for use in tumor models in rats.

## Results

All animals could be treated as planned and were included in the results. During anesthesia no complications were observed. The usage of anesthesia antagonists resulted in a fast and smooth recovery from anesthesia.

A small amount of reflux was observed twice after footpad injection. However, the primary tumor could be induced in all cases over time. Differences were noticed regarding their speed of development with earlier signs in the hock injection group.

**Fig. 1:**
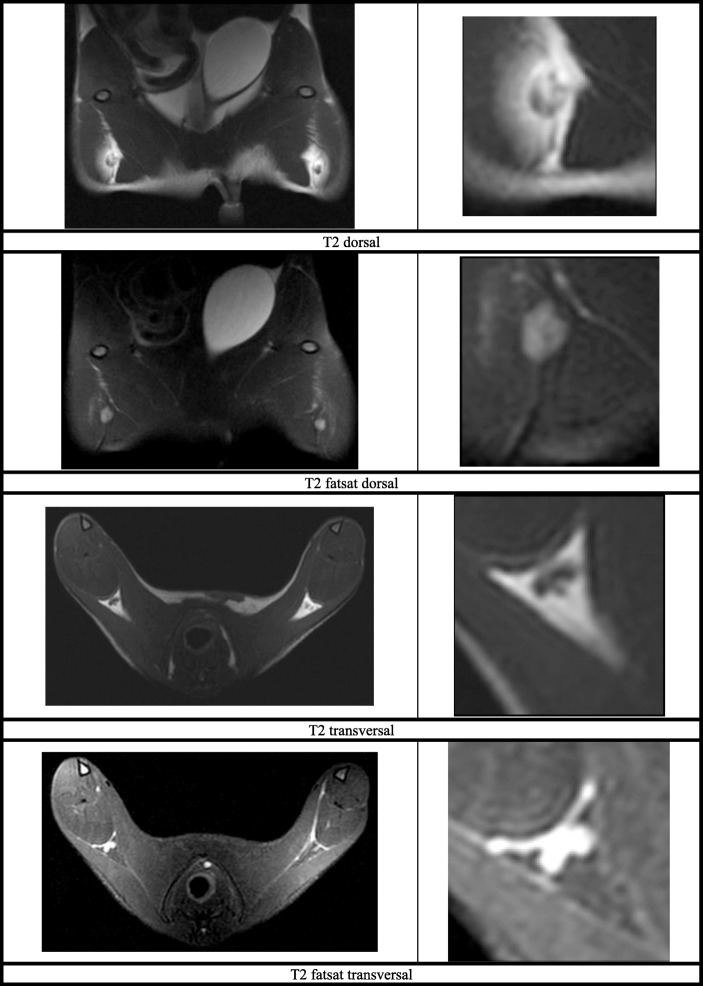
**a**-**d**) photographs of footpad injection (FP) - primary tumor (right) in comparison with control side (left); **a**): footpad injection; **b**): primary tumor after FP (21 days); **c**): primary tumor after FP (21 days); **d**) control side after NaCl FP (21 days)**. e**-**h**) photographs of Hock injection (Hock) - primary tumor (right) in comparison with control side (left); **e**): Hock injection; **f**): primary tumor after Hock injection (18 days); **g**) control side after NaCl Hock injection (18 days); **h**): primary tumor after Hock injection without fur (18 days)

In the first 5 days after inoculation tumor development could not be observed, neither for footpad nor for the hock injection technique. Thereafter, onset of tumor development could be clinically observed in the hock injection group at 7 days after tumor inoculation compared with 10 days after footpad injection. The spread of the tumor was limited to the surrounding soft tissue for both groups. The fast growing tumor in the hock injection group was palpable under the skin and was located at the injection site. At eight days after tumor inoculation at the hock, all animals developed a distinct primary tumor. In the footpad group, a distinct primary tumor developed 11 days after injection, which was like in the hock injection group located and limited to the injection site (swollen foot). (Figure 1).

The maximum survival time was reached after 18 days in the hock injection group and after 21 days in the footpad injection group. In both cases animals were unable to put weight on their injected limbs.

The imaging protocol proved suitable in all cases and confirmed growth of the primary tumor while it also enabled monitoring of popliteal lymph nodes prior to organ sampling. Fat suppression techniques allowed visualizing lymph nodes without surrounding fat and documenting their size.

All primary tumors identified by MRI where confirmed macroscopically during organ sampling and later also histopathologically. Based on MRI images, the size of both popliteal lymph nodes was measured in width and length before tumor inoculation (first MR) and directly before euthanasia (second MR). The measured size of the popliteal lymph nodes on the tumor-bearing limb (right side) differed significantly between the first and the second MRI (width right: *p* = 0.000, length right: *p* = 0.008). Splitting the data by type of injection showed significance between MRI examinations on the right side after hock injection (width right: *p* = 0.009, length right: *p* = 0.041). All results are summarized in Table 1.

**Table 1:** Descriptive data analysis and Mann-Whitney U-Test between 1st and 2nd MRI, *significant p < 0.05 [Median (Min, Max, *p*-value)]

Side		Lenght	Width
right		3.06 (2.28, 5.73, *p* = 0.008)*	2.19 (1.58, 3.66, *p* = 0.000)*
left		2.88 (2.03, 3.28, *p* = 0.932)	2.02 (1.52, 2.59, *p* = 0.932)
Type of injection	Side	Lenght	Width
FP	right	2.99 (2.29, 5.62, *p* = 0.132)	2.15 (1.58, 3.22, *p* = 0.065)
FP	left	2.84 (2.45, 3.20, *p* = 1.000)	1.99 (1.52, 2.30, *p* = 0.699)
Hock	right	3.09 (2.28, 5.73, *p* = 0.041)*	2.25 (1.68, 3.66, *p* = 0.009)*
Hock	left	2.88 (2.03, 3.28, *p* = 0.937)	2.09 (1.74, 2.59, *p* = 0.937)

Macroscopically the primary tumors after hock injection were larger in size. They strongly compressed adjacent tissue and exhibited more necrosis and also hemorrhage compared to the primary tumor after footpad injection.

**Fig. 2:**
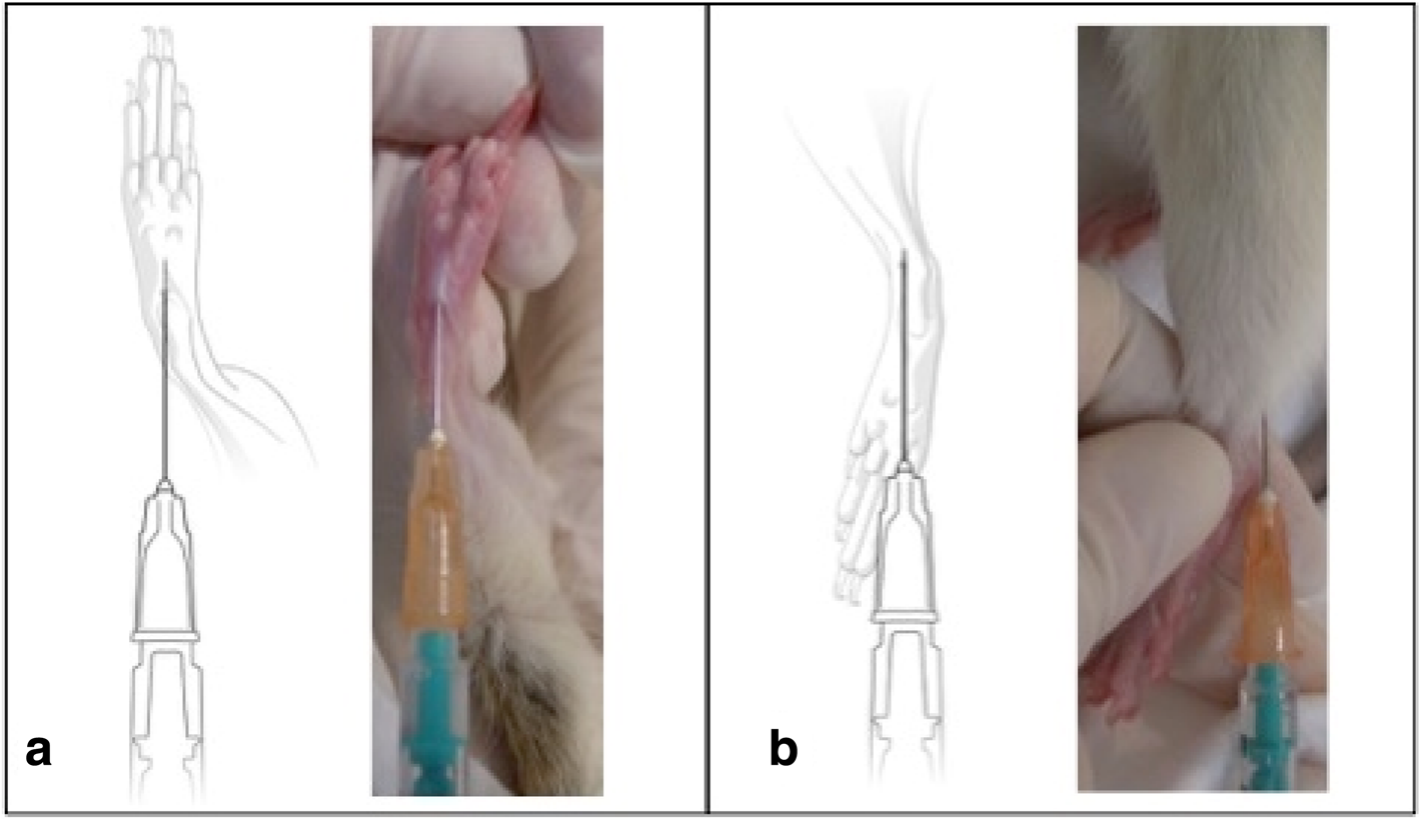
**a**-**h**) Histological examples of tumor phenotype and immunohistochemical reactions

Histopathologically the primary tumors could be identified as a high-grade malignant adenocarcinoma independent of injection site, with the same morphology score (mean_FP_: 2.50 ± 0.71, mean_Hock_: 2.50 ± 0.71). The samples of the primary tumors showed all typical signs of malignancy (mitoses, cellular atypia, cellular apoptosis, presence of vascular invasion, hemorrhage, necrosis, compression of adjacent tissue). (Figure 2).

All other organs were structurally unremarkable (HE). Metastases in distant organs (lungs, liver, spleen, kidneys, adrenals, muscle) could not be detected, neither during MRI examination nor later during screening the histological sections (HE). Scoring of structural changes in popliteal lymph nodes (HE) reached slightly higher scores in the tumor bearing right leg (mean_FP_: 1.25 ± 0.83, mean_Hock_: 0.50 ± 0.50) compared with the left control side (mean_FP_: 1.00 ± 0.71, mean_Hock_: 0.25 ± 0.43). Additional IHC (Lu-5, Galectin 3, PSA) was negative for tumor cells and showed unspecific reaction with staining of normal structures: macrophages, plasma cells and some tissue background. Although lymph nodes showed a middle to severe a reactive tissue structure, early lymph node tumor metastasis could not be observed macroscopically nor detected histologically (no tumor nests or single tumor cells).

## Discussion

For animal studies in rodents, tumor induction is a widely used technique to observe tumor biology [7], treatment [8] or metastases [9]. Tumor growth and the behavior of metastases are of primary interest for clinical diagnosis and treatment planning. This study evaluated the feasibility of the MatLyLu tumor model by using two type of injections in Copenhagen rats, the hock or footpad injection. Although animals of both groups developed a manifest primary tumor after inoculation, differences could be detected in time of tumor development.

Tumor induction differences could be reproduced between part 1 and part 2 of this study and showed that the injection type is of importance. Hock injection leads to a faster growth of the primary tumor, whereas footpad injection needed 3 days longer until manifestation of the primary tumor. In addition, hock injection allowed the primary tumor to develop bigger in size compared to footpad injection. The very tight structure and limited amount of surrounding tissue in the animal’s footpad could explain this. Instead the hock injection offers a larger subcutaneous space for inoculation with higher volumes and still reduced risk for reflux. Especially studies with small injection volumes could profit from that experience, as it is important to avoid reflux in order to minimize experimental error and variability of the data observed.

At the same time, the location of injection has a strong influence on animal welfare. Hock injection was already applied to study local immune responses in sheep [10–12] and mice. [5] In these studies it could be observed, that the level of discomfort was much lower due to tumor growth on a not-weight bearing area. Any animal model associated with a fast growing primary tumor may profit from the use of the hock injection, because thereby no weight bearing structure is involved. Footpad injection is always related to a primary lesion on the weight bearing feet, which results in reduced animal welfare during tumor development. [5] Systemic limitations after footpad injection regarding food uptake, activity, mobility or weight loss were not observed in the initial stage until clinical signs of tumor manifestation. However, at a later stage of tumor growth clinical relevant immobility occurred within 2 days for both types of injections with the first day showing low grade lameness and on the second day non-weight bearing of the affected limb. This limited the maximum survival time after hock injection to 18 days compared to 21 days after footpad injection. While on one hand, footpad injection reduces animal welfare during early tumor development due to tumor growth on the weight bearing hind leg, it allows prolongation of the maximum survival time on the other hand. This may be explained by the anatomical related limitations of the tumor growth and size at the animal’s foot pad.

This difference of tumor size and growth could be advantageous for specific research questions where time and size play a role as parameters. In any case this study showed that both types of injection will be able to reliable induces a primary Matlylu primary tumor.

Secondary to the primary tumor, lymph node involvement and metastasis are of interest while studying tumor models. Lymphatic drainage is already described for Matlylu. [13] On this account, lymph node involvement was also observed in the current study during MRI examination and was verified by histological evaluation. A significant and remarkable increase of lymph node size could be detected for popliteal lymph nodes (1st/2nd MRI _size popliteal width right_: *p* = 0.000, 1st/2nd MRI _size popliteal length right_: *p* = 0.008). As they are the sentinel lymph nodes of the hind limb and both are close to both injection sites, it was expected to induce lymph node metastasis of the primary Matlylu tumor. [14–16] Based on MRI findings the reason for enlargement could not be differentiated between reactive or metastatic. The imaging protocol used in this study was optimized for the available 3 Tesla MRI. Although this kind of MRI is originally not produced for rodents images of a very good quality were obtained using the settings described above. The imaging protocol allowed recognizing the primary tumor as well as normal and enlarged popliteal lymph nodes. Two ROI were used to optimize settings for increased image quality. One focus was set caudally of the pelvis to visualize the primary tumor and popliteal region, the other focus was set for a whole body scan of the rat to check for metastasis in other organs such as liver, lungs, brain. Measurements of lymph node size were based on DICOM images and limited by the small size of the lymph nodes, which were visible in two to three slices only.

Histological evaluation of the lymph nodes should help to clarify the reason for enlargement (reactive or metastatic). However, neither HE staining nor IHC staining were able to detect early lymph node metastases (single-cell and micro-metastases). IHC showed an unspecific reaction and some tissue background. Based on the fact that primary tumors between 5th and 21st day were evaluated, we assume that this may be at least partially explained by insufficient lymph node involvement in early stages. But then, the lymph nodes of a later stage primary tumor did not show either any signs of metastatic changes. No differences between injection types were observed regarding metastatic lymph node involvement. Based on that finding it could be concluded that early stage lymph node involvement could not be detected within reactive changes by using histology. These findings would go along with earlier studies published where it was suggested that the site of tumor inoculation and circulatory anatomy does not influence the pattern of metastasis development in organs. [17]

As limitations of the study the small number of animals and asymmetric study design (between part 1 and part 2) has to be considered. Additionally, neither HE staining nor IHC staining were able to detect early lymph node metastases. The conflict between rapid tumor growth/metastatic activity and animal welfare influenced the possibility to observe lymph nodes of a later stage. Since animals after hock injection have a shorter survival time, researchers using this model will be limited to a smaller timeframe to study results of interest. Nevertheless, in both, the animal model with hock and foot pad injection, Matlylu tumors could be induced and serve as animal model for further investigations of prostate tumors.

## Conclusion

In conclusion hock injection is a feasible alternative technique compared with footpad-injection in rats. It provides a save and easy injection method for various early-terminated applications with the potential to increase animal welfare during tumor models in rats.

## Methods

### Animals

In the study healthy female Copenhagen rats were used, which were all research animals. In the first part of the study 12 animals with a Mean ± SD age of 103 ± 10.8 days (range 94 to 127 days) and a Mean ± SD body weight of 162 ± 10.6 g (range, 148 to 180 g) and in a second part of the study 4 animals with a Mean ± SD age of 90.5 ± 4.2 days (range 86 to 95 days) and a Mean ± SD body weight of 151 ± 8.0 g (range, 144 to 161 g) were used. After delivery, the animals had seven days of acclimatization. All animals had free access to food and water and were placed at a regular light/dark cycle (7:00 a.m. to 07:00 p.m., light) and a controlled temperature (20–23 °C). The animals were trained and accustomed to human handling to reduce stress during the study. All animals were kept in an open cage system, group housed (2 animals together) in type T2000 cages (LxBxH 610x435x215mm). Single housing of animals was not necessary, with exception of the last animal of the cage (for maximum 3 days). During acclimatization the rats were handled every day to make them familiar with the research personnel (stress reduction). Once a day, and after tumor inoculation twice a day, all animals were checked using a health-check-protocol. The health check included assessment of mobility, posture, pelt, eyes, social behavior and weight loss using a specially developed score sheet for assuring animal well being. In the first part of the study one animal of each group (hock vs footpad injection) was observed over a specific tumor developmental time of 5, 9, 11, 14, 15, 18 and 21 days. In the second part of the study the tumor developmental time was set at 9, 11, 13 and 15 days depending on the clinical observations. (Table 2) The tumor reached its maximum developmental time once the animal was stopped weight bearing on the injected hind limb.

**Table 2:** Table showing study design and allocated groups

Study part	Animal	Type of Injection [hock = H, FP = footpad]	Tumor developmental time [days]
1	1	H	5
	2	FP	5
	3	H	9
	4	FP	9
	5	H	11
	6	FP	11
	7	H	14
	8	FP	14
	9	H	15
	10	FP	15
	11	H	18
	12	FP	21
2	13	H	9
	14	FP	11
	15	H	13
	16	FP	15

### Anaesthesia

General anesthesia was necessary for imaging as well as for a safe and standardized tumor inoculation. The induction of anesthesia was performed by subcutaneous injection of a drug mixture (1 ml/kg BW), containing 1 ml fentanyl (0.05 mg/ml) with 4 ml midazolam (5 mg/ml), 1.5 ml medetomidine (1 mg/ml) and 3.5 ml of 0.9% NaCl. During time of anesthesia the eyes were covered with ointment (Vitamin A). Anesthesia state was maintained by subcutaneous injection of the same drug mixture using 0.5 ml/kg BW every 30–60 min. Anesthesia was terminated by reversing action of the used anesthesia drugs. For this purpose a drug mixture was prepared of 3 ml naloxone (0.4 mg/ml), 20 ml flumazenil (0.1 mg/ml), 1.5 ml atipamezole (5 mg/ml) and 5.5 ml of 0.9% NaCl and administered intraperitoneally at 3 ml/kg BW.

### Imaging

**Fig. 3:**
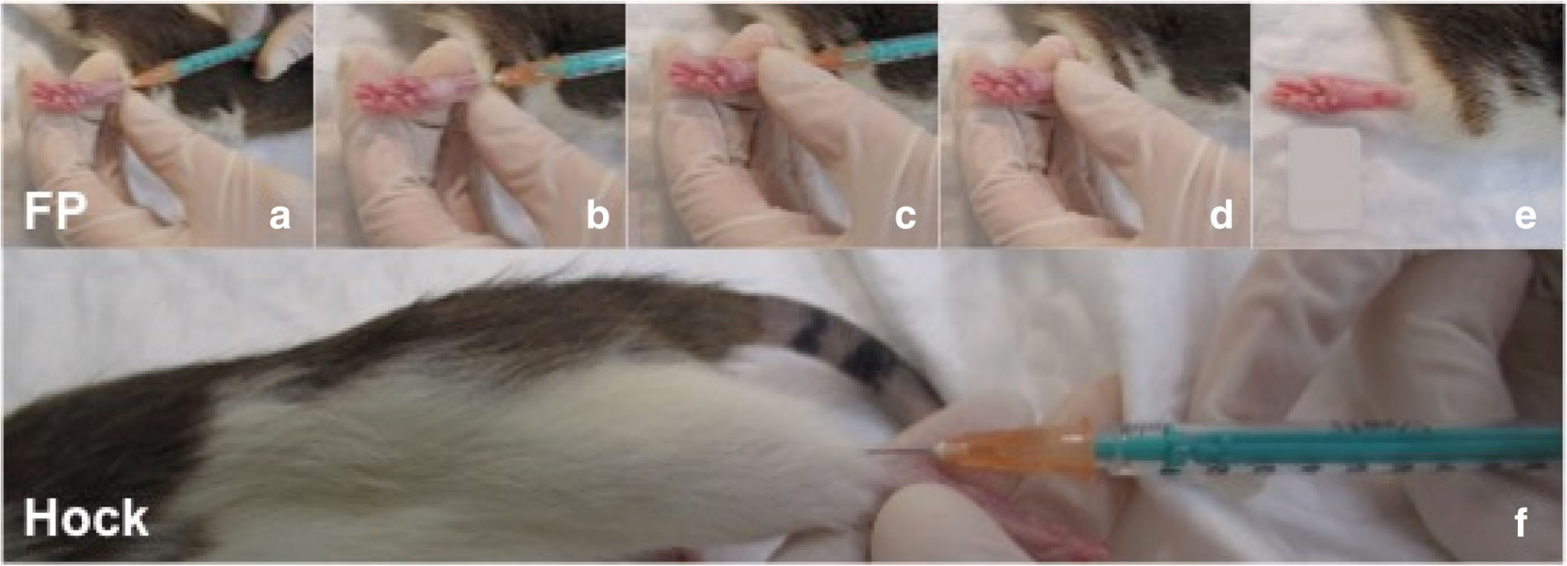
MRI Images of Copenhagen rats - pelvic region (left image) and right popliteal lymph node in detail (right image): T2 dorsal; T2 fatsat dorsal; T2 transversal; T2 fatsat transversal; T1 transversal; T1 SPIR transversal

All MRI examinations were performed with a 3.0 Tesla scanner with the rats under general anesthesia. During examination animals were positioned in dorsal recumbency with the head towards the gantry. Imaging protocols were adapted over time and used two regions of interest (ROI). First ROI focused on the hind limbs and included the primary tumor as well as popliteal lymph nodes of both sides. The second ROI focused on the whole body of the rats and included thorax and abdomen. The acquired scan protocol contained T1 and T2 weighted sequences (Fig. 3). Two different coils^l,m^ were used for acquisition of the data (3 T Dual Microscopy coil 47 mm and M 16c-TR-KneeCoil). During MRI, the rats got earplugs (cotton ball) for noise and stress reduction. The first MRI examination was performed directly before tumor cells were inoculated. The second MRI examination was performed after an animal dependent tumor development time (5th to 21st day) and followed by euthanasia and organ sampling. After their second MRI all animals were euthanized, while being deeply anesthesized, with 0.5 ml/kg BW Potassiumchloride intracardially (> 2 mmol/kg KCl). Based on MRI images the size of lymph nodes was measured in width and length.

### Injections

In this study 1 × 10^6^ MatLyLu tumor cells solved in 0.1 ml NaCl were injected into 16 Copenhagen rats. Required tests confirmed that the cells were pathogen free (Additional file 2). They were stored at − 80 °C prior use.

In the first part of the study cells were injected immediately after being thawed. The thawed cell suspension (1 ml) was transferred into a falcon tube, containing 10 ml NaCl at 37 °C. After centrifuging (150 g for 5 min), the supernatant was removed and resuspended in 300 μl NaCl. The cells were drawn into the syringe using a 18 Gauge needle to minimize shear stress.

**Fig. 4:**
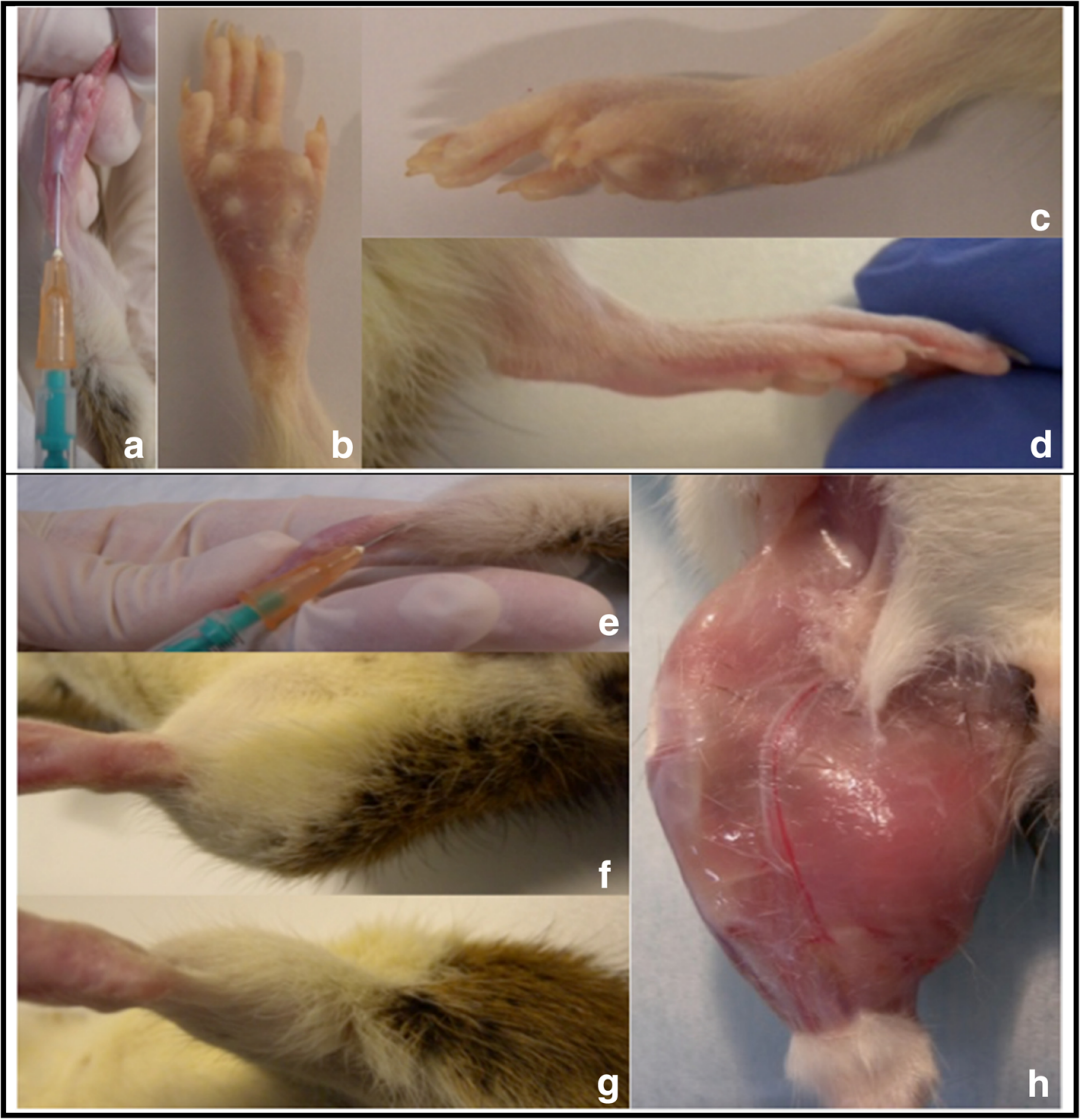
**a** Foot-pad (FP) and **b**) Hock Injection - both shown as cartoon and photograph

**Fig. 5:**
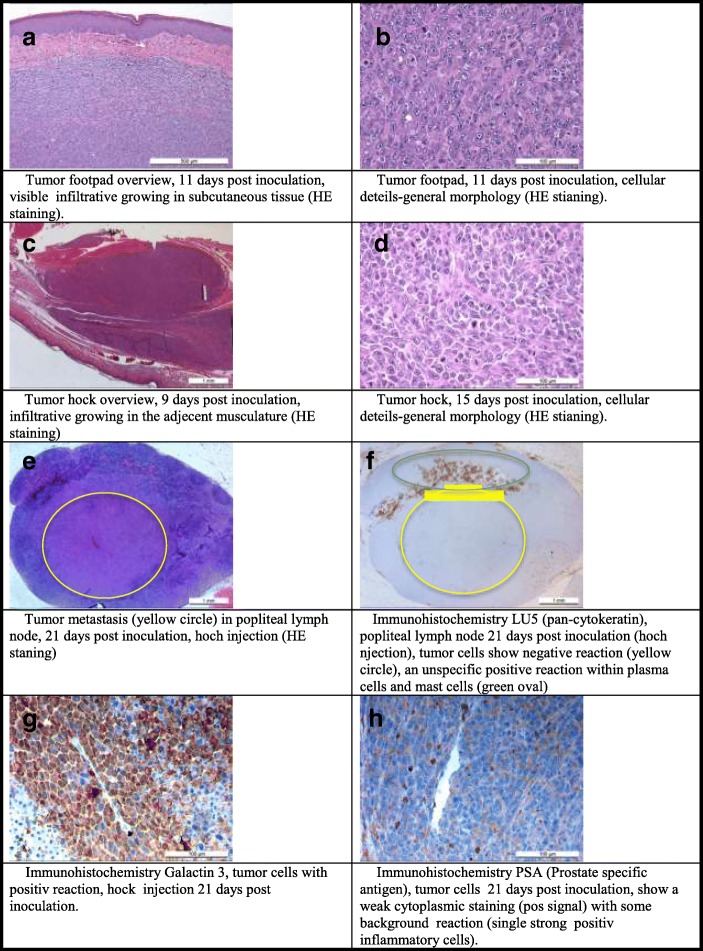
**a**-**e**) Series of images during footpad injection technique (FP); **f**) photograph of Hock injection technique

In the second part of the study, MatLyLu cells were cultured at 37 °C in a humidified incubator (5% CO2) in RPMI 1640 medium (containing 2 mM Glutamine), supplemented with 10% fetal bovine serum, 250 nM Dexamethasone and 1% penicillin/streptomycin for 7 days prior to injection. Every second day cells were split 1:20 into a fresh cell culture flask. Prior to in vivo injection, medium was changed to NaCl and the cell suspension of 1.1 × 106 cells/100 μl was prepared for injection. One group (8 animals) was injected into the footpad according to the “Footpad Injections Guidelines in Mice and Rats” published by IACUC. [18] The other group (8 animals) received a hock injection, which was located at the lateral tarsal region just above the ankle. [5] (Fig. 4) The tumor cell injections (0.1 ml) were conducted subcutaneously with a 25G needle into the right hind limb, while using the left hind limb as individual control. As control 0.1 ml 0.9% NaCl was subcutaneously injected. All injections were done under general anesthesia after MRI examination. During injection the needle inserted bevel was turned towards the skin and the mixture was injected by slow but firm pressure on the syringe plunger. After both injections, a small “bubble” of mixture was seen subcutaneously at the injection site. At the time of removing the needle, gentle pressure was applied to prevent leakage (reflux) (Fig. 5).

### Histopathology

Directly after the second MRI examination, the rats were euthanized and dissected. The following organs were collected and prepared for histopathology: brain, heart, right middle lung lobe, liver, spleen, kidneys, adrenal glands, lymph nodes (mediastinal, inguinal, popliteal), muscle (quadriceps femoris) and the primary tumor. Slides of all samples were stained with hematoxylin and eosin (HE). Additionally immunohistology was performed with the aim to identify adenocarcinoma cells within primary tumor and to identify possible metastasis in lymph nodes. Serial sections were deparaffinized, heat-mediated antigen retrieval was conducted (95 °C, 20 min) and sections were incubated in room temperature overnight with following primary antibodies: Pan-Cytokeratin, PSA and Galactin-3 (Table 3). Secondary anti-mouse IgG was applied in (1:200 dilution) for 30 min followed by the Amino-9-ethyl-carbazole (AEC) substrate kit as a chromogen. Finally the sections were counter-stained with Gill’s hematoxylin for 3 min and cover-slipped with an aqueous mounting media.

**Table 3:** Table showing detailed antibody informations

Marker	Provider	Order Number	Dilution	Read-out
Lu5 (Pan-cytokeratin)	Abcam	ab27988	1:10	Cells of epithelial origin (carcinoma cells)
PSA	Abcam	ab53774	1:500	Anti-Prostate Specific Antigen
Galactin-3	Abcam	ab2785	1:100	Adenoma and carcinoma cells (incl MatLyLu cells (Pienta et al. 1995)

All slides were examined and semiquantitatively evaluated (Table 4).

**Table 4:** Table showing semiquantitative scoring for histopathology examination

Organ	Scoring	Description
Liver	0	–
	1	mild focal fatty infiltration around central vein(s)
	2	mild to moderate fatty infiltration around central vein(s) and in portal area, sinusoid dilatation
	3	severe fatty infiltration around central vein and in portal area, necrosis (hepatocellular dissociation)
Spleen	0	–
	1	activated follicles, increased hemosiderosis
	2	increase of red and white pulp (follicles etc.),
	3	neutrophil granulocytes in red pulp, necrosis, fibrosis
Lymphnodes	0	–
	1	secondary follicle activation, cortex vascular lesions (dilation of blood and/or lymphatic vessels)
	2	Localized: Secondary follicle activation, follicular depletion (atrophy), lymphatic sinus ectasia,medullar and cortex vascular lesions (dilation of blood and/or lymphatic vessels), macrophage hyperplasia (cortical-sinus)
	3	Whole organ (Cortex, Paracortex, Medulla): Secondary follicle activation, follicular depletion (atrophy), lymphatic sinus ectasia,medullar and cortex vascular lesions (dilation of blood and/or lymphatic vessels), macrophage hyperplasia (cortical and medullary sinus)
Kidneys	0	–
	1	single protein liquid in proximal tubuli
	2	generalized protein liquid in proximal tubuli, interstitial nephritis, swollen mesangial cells
	3	necrosis, tubulonephrose, atrophic glomeruli, tubular cysts, papillar necrosis, fibrosis
Brain	0	–
	1	severe hyperemia and congestion, oedema (widen “Virchof-Robin” space)
	2	additional gliosis, single neuron necrosis (shrunken hypereosinophilic neurons)
	3	additional severe gliosis/astrocytosis, neuronal necrosis, encephalitis, status spongiosus (severe oedema), lost of myelin sheath (demyelination)
Lung	0	–
	1	severe hyperemia and congestion, alveolar oedema (liquid in alveoli with cellular reaction)
	2	additional some inflammatory cells in alveoli/interstiitum (macrophages, lymphocytes, neutrophils)
	3	additional multiple inflammatory cells in alveoli/interstiitum (macrophages, lymphocytes, neutrophils), proliferation of bronchial epithelium, bronchitis
Heart	0	–
	1	severe hyperemia and congestion
	2	additional single perivascular inflammatory infiltrates and degeneration of myocytes
	3	additional myocard necrosis (focal and multifocal), deposits within vascular wall
Adrenal gland	0	–
	1	severe hyperemia and congestion
	2	mild cortex/medulla activation (nodular hyperplasia), hypertrophy
	3	severe cortex/medulla activation (nodular hyperplasia), hypertrophy
Muscle	0	–
	1	severe hyperemia and congestion, interstitial oedema, mild satelitosis
	2	additional swelling of the myocytes, degeneration (centraliation of myocytes nuclei), focla loss of striation, hypereosinophilic myocytes
	3	additional necrosis, inflammation, fatty infiltration
Tumor	0	–
	1	tumor focus in primary organ with local invasion (infiltrative growth)
	2	tumor focus in primary organd with invasion and compression of adjacent tissue, mitosis per HPF40x (1–2), metastases in local lymph node (popliteal LNN)
	3	mitosis per HPF40x (3–7), vascular invasion (lymphatics, blood vessels), necrosis, cellular apoptosis, metastases lymph nodes (additional to popliteal Lnn) and other organs

### Statistic

Statistical analysis was performed with SPSS. Descriptive analysis (median, min, max) was performed for continuous variables. Taking into account that the number of examined rats was limited, the population distribution was not assumed to be normal. Consequently, nonparametric rank sum tests (Mann-Whitney U-test) were used to assess differences between groups. For all values *p* < 0.05 was considered to be significant.

## Additional files

Additional file 1: Score sheet for animal health. (PDF 51 kb)

Additional file 2: Pathogen cell tests. (PDF 41 kb)

## Abbreviations

3R: Replace, reduce, refine
HE: Hematoxylin and eosin
IHC: Immunohistochemistry
MatLyLu: Metastatic adenocarcinoma tumor with metastasis in lymph nodes and lungs
MRI: Magnetic resonance imaging
NaCl: Natrium-chlorid
PSA: Prostate specific antigen
ROI: Region of interest
