# Supplementary material for: Comparison of hock- and footpad-injection as a prostate adenocarcinoma model in rats
Source: BMC Vet Res. 2018 Nov 6;14:327. doi: 10.1186/s12917-018-1659-x (PMC6219108; doi:10.1186/s12917-018-1659-x)
Supplement: Supplementary file 1 — Score sheet for animal health. (PDF 51 kb) [file 12917_2018_1659_MOESM1_ESM.pdf]

Daily observation score sheet

**Behavior**	**Score**	**Description**
Mobility	3	normal
	2	hesitant
	1	Only if stressed
Weight bearing	3	normal
	2	reduced
	1	no weight bearing
Space occupying lesion	3	no
	2	mild
	1	severe
Posture	3	normal
	2	stocky
	1	Hump/craned
Pelt	3	normal
	2	faint, porphyrin deposition
	1	Max. faint, scruffy/ piloerection
Eyes	3	normal
	2	porphyrin rings
	1	Porphyrin rings/ vitreus, clotted
OR wound	3	dry
	2	Sutures partially open, reddish
	1	gapping, reddened
Social behaviour	3	normal
	2	isolated, aggressive
	1	apathetic, isolated
Loss of weight	3	<5%
	2	<10%
	1	>10%

Inform Study Director if:

Post operative	Any subscore 1 or 2
	Persisting score 2 for more than 2 days post operative
Non-operated	Any total score <27
Direct Euthanasia if	After consulting the study director: Score <18
