# Supplementary material for: Comparison of hock- and footpad-injection as a prostate adenocarcinoma model in rats
Source: BMC Vet Res. 2018 Nov 6;14:327. doi: 10.1186/s12917-018-1659-x (PMC6219108; doi:10.1186/s12917-018-1659-x)
Supplement: Supplementary file 2 — Pathogen cell tests. (PDF 41 kb) [file 12917_2018_1659_MOESM2_ESM.pdf]

PCR evaluation	MatLyLu PMAG
Ectromelia	negative
EDIM	negative
Hantaan	negative
LCMV	negative
LDEV	negative
MAV 1	negative
MAV 2	negative
mCMV	negative
MHV	negative
MNV	negative
Mycoplasma pulmonis	negative
Mycoplasma sp.	negative
MVM	negative
MPV	negative
Polyoma	negative
PVM	negative
REO 3	negative
Dendai	negative
TMEV	negative
KRV	negative
RMV	negative
RPV	negative
H1	negative
RCV/SDAV	negative
RTV	negative
